# The European Multidisciplinary Evidence‐Based Guideline on Pancreatic Cancer: Methodological Protocol

**DOI:** 10.1002/gin2.70074

**Published:** 2026-07-13

**Authors:** Laura C. Leeuwenburgh, Magdalena Holze, Dena Akhoundzadeh, Adnan Alsourani, Ricardo Alvarez Jimenez, Paul C. M. Andel, Charlotte Baggerman van Houweninge, Alberto Balduzzi, Caro L. Bruna, Fabio Casciani, Alice Cattelani, Aniek E. van Diepen, Heleen Driessens, Daphne H. M. Droogh, Jeska A. Fritzsche, Eline van Gansewinkel, Luana Genova, Anne M. Gehrels, Gonzalo Gómez Dueñas, Riccardo Guastella, Lis S. M. Hoeijmakers, Tijs J. Hoogteijling, Ammar A. Javed, Berk Kaan Aktas, Benedict Kinny‐Köster, Amber Lamoré, K. Seng Liem, Gabriella Lionetto, Lorenzo Lo Faro, Lisanne Lutter, Manuela Mastronardi, Carlo H. Maurer, Roberto Montorsi, Julian Musa, Ingmar F. Rompen, Jacobien C. M. Scheepens, Dana Sochorová, Thomas F. Stoop, Nuray Tezcan, Bas A. Uijterwijk, Roberta Vella, Federica Vernuccio, Rogier P. Voermans, Mohammad Abu Hilal, Volkan Adsay, Hana Algül, Valentina Ambrosini, Livia Archibugi, Eva Backman, Sorin T. Barbu, Ugo Boggi, Charlotte Borge‐Andersen, Stefan Kobbelgaard Burgdorf, Alice Clarkson, Eric van Cutsem, Pieter‐Jan Cuyle, Lois A. Daamen, Peter De Rosa, Marcel den Dulk, Celia Diez de los Rios, Amanda Drury, Irene Esposito, Simon M. Everett, Arantza Farina Sarasqueta, Isabella Frigerio, Bas Groot Koerkamp, Thilo Hackert, Florence Huguet, Nikolaos Kartalis, Anne Letsch, Pex Langenberg, Denny Levett, Gabor Liposits, Núria Malats, Hannah van Malenstein, Giuseppe Malleo, Zorana Maravic, Giovanni Marchegiani, Ana Martins, Martijn R. Meijerink, Christoph W. Michalski, J. Sven D. Mieog, Eleni Moka, Somnath Mukherjee, Cristina Nanni, Franco Orsi, Demetris Papamichael, Stefano Partelli, Cathy Payne, Ilaria Pergolini, Dejan Radenkovic, Keith J. Roberts, Roberto Salvia, Alain Sauvanet, Marta Scorsetti, Matthias Seebo, Thomas Seufferlein, Stefan Stättner, Geoffroy Vanbiervliet, Caroline S. Verbeke, Thomas Volk, Jens Werner, Johanna W. Wilmink, Marc Zins, Thierry Conroy, Per Pfeiffer, Michel Ducreux, Mahsoem Ali, Miranda Langendam, Yasaman Vali, Elena B. Rangelova, Marc G. Besselink, for the Multi‐Societal European Evidence‐Based Guideline on Pancreatic Cancer

**Affiliations:** ^1^ Amsterdam UMC, location University of Amsterdam Department of Surgery Amsterdam the Netherlands; ^2^ Cancer Center Amsterdam Amsterdam the Netherlands; ^3^ Department of General, Visceral and Transplantation Surgery Heidelberg University Hospital Heidelberg Germany; ^4^ University of Verona Verona Italy; ^5^ Unit of Pancreatic Surgery University of Verona Hospital Trust Verona Italy; ^6^ General Surgery Department Hospital General Universitario Gregorio Marañón Madrid Spain; ^7^ Amsterdam UMC, Palliative and Pain Medicine Department of Anesthesiology Amsterdam the Netherlands; ^8^ Department of Surgery, Regional Academic Cancer Center Utrecht UMC Utrecht Cancer Center and St. Antonius Hospital Nieuwegein Utrecht the Netherlands; ^9^ General Surgery Unit Pederzoli Hospital, Peschiera del Garda Verona Italy; ^10^ Department of Medical Oncology, Maastricht University Medical Centre Maastricht University Maastricht the Netherlands; ^11^ Department of Surgery, Division of Hepato‐Pancreato‐Biliary Surgery and Liver Transplantation University Medical Center Groningen Groningen the Netherlands; ^12^ Department of Surgery Leiden University Medical Center Leiden the Netherlands; ^13^ Amsterdam UMC, location University of Amsterdam Department of Gastroenterology and Hepatology Amsterdam the Netherlands; ^14^ Department of Internal Medicine Maastricht University Medical Center Maastricht the Netherlands; ^15^ Pancreatic Surgery Unit, Pancreas Translational and Clinical Research Center IRCCS San Raffaele Institute Milan Italy; ^16^ Vita‐Salute San Raffaele University Milan Italy; ^17^ Amsterdam UMC, location University of Amsterdam Department of Medical Oncology Amsterdam the Netherlands; ^18^ General and Digestive Surgery Department Reina Sofia University Hospital Cordoba Spain; ^19^ Hepato‐Pancreato‐Biliary and Liver Transplant Surgery Unit, Department of Surgical,Oncological and Gastroenterological Sciences Padua University Hospital Padua Italy; ^20^ Department of Surgery, NUTRIM Institute of Nutrition and Translational Research in Metabolism Maastricht University Maastricht Netherlands; ^21^ Department of Surgery The NYU Grossman School of Medicine and NYU Langone Health New York City New York USA; ^22^ Department of Pathology KOC University Istanbul Turkey; ^23^ Amsterdam University Medical Center Department of Radiology and Nuclear Medicine Amsterdam the Netherlands; ^24^ Department of Gastroenterology and Hepatology Leiden University Medical Center Leiden the Netherlands; ^25^ Department of Radiotherapy and Radiosurgery IRCCS Humanitas Research Hospital Milan Italy; ^26^ Amsterdam UMC, location University of Amsterdam Department of Pathology Amsterdam the Netherlands; ^27^ Division of General Surgery, Department of Medical, Surgical and Health Sciences University of Trieste Trieste Italy; ^28^ Department of Internal Medicine II, Klinikum rechts der Isar, TUM School of Medicine and Health Technical University of Munich Munich Germany; ^29^ Department of General, Visceral, Thoracic, and Transplant Surgery University Hospital Giessen and Marburg Giessen Germany; ^30^ Department of Radiation Oncology University Medical Center Utrecht Utrecht the Netherlands; ^31^ Department of Surgery Tomas Bata Regional Hospital Zlin Czech Republic; ^32^ Department of Hepato‐Bilio‐Pancreatic Surgery Pederzoli Hospital, Peschiera del Garda Verona Italy; ^33^ Precision Medicine in Medical, Surgical, and Critical Care, (Me.Pre.C.C.) University of Palermo Palermo Italy; ^34^ Section of Radiology, Department of Biomedicine, Neuroscience and Advanced Diagnostics (BIND) University of Palermo Palermo Italy; ^35^ Department of Surgery, School of Medicine the University of Jordan Amman Jordan; ^36^ Department of Surgery University Hospital Southampton NHS Foundation Trust Southampton UK; ^37^ Comprehensive Cancer Center Munich TUM, TUM University Hospital, Klinikum rechts der Isar Technical University of Munich Munich Germany; ^38^ Institute for Tumour Metabolism, TUM University Hospital, Klinikum rechts der Isar Technical University of Munich Munich Germany; ^39^ Nuclear Medicine Alma Mater Studiorum University of Bologna Bologna Italy; ^40^ Nuclear Medicine, IRCCS Azienda Ospedaliero‐Universitaria di Bologna Bologna Italy; ^41^ Pancreato‐Biliary Endoscopy and Endosonography Division, Pancreas Translational and Clinical Research Center San Raffaele Scientific Institute IRCCS Milan Italy; ^42^ PALEMA Cancer Society Stockholm Sweden; ^43^ Department of Surgery University of Medicine and Pharmacy “Iuliu Hatieganu” Cluj‐Napoca Romania; ^44^ Division of General and Transplant Surgery University of Pisa Pisa Italy; ^45^ Pankreaskreft Nettverk Norge Oslo Norway; ^46^ Department of Surgery Rigshospitalet Copenhagen Denmark; ^47^ Pancreatic Cancer UK London UK; ^48^ Department of Digestive Oncology University Hospitals Leuven and KU Leuven Leuven Belgium; ^49^ Department of Gastroenterology and Digestive Oncology Imelda General Hospital Bonheiden Belgium; ^50^ Division of Imaging and Oncology University Medical Center Utrecht Utrecht the Netherlands; ^51^ Department of Surgery Maastricht University Medical Center Maastricht the Netherlands; ^52^ School of Nursing, Faculty of Medicine and Health Sciences Barcelona University Barcelona Spain; ^53^ School of Nursing, Psychotherapy and Community Health Dublin City University Dublin Ireland; ^54^ Institute of Pathology Heinrich‐Heine University and University Hospital Düsseldorf Germany; ^55^ Department of Gastroenterology Leeds Teaching Hospitals NHS Trust Leeds UK; ^56^ Collegium Medicum SAN University Lodz Poland; ^57^ Department of Surgery Erasmus MC Cancer Institute Rotterdam the Netherlands; ^58^ Department of General, Visceral and Thoracic Surgery University Medical Center Hamburg‐Eppendorf Hamburg Germany; ^59^ Department of Radiation Oncology, Tenon Hospital AP‐HP, Sorbonne University Paris France; ^60^ Department of Clinical Science, Intervention and Technology Karolinska Institutet Stockholm Sweden; ^61^ Department of Radiology Karolinska University Hospital Stockholm Sweden; ^62^ Department of Haematology and Oncology, University Cancer Center Schleswig‐Holstein University Medical Center Schleswig‐Holstein Kiel Germany; ^63^ Living With Hope Utrecht the Netherlands; ^64^ NIHR Southampton Biomedical Research Centre University Hospital Southampton NHS Foundation Trust/University of Southampton Southampton UK; ^65^ Department of Oncology Innlandet Hospital Trust, Division Gjøvik‐Lillehammer Gjøvik Norway; ^66^ Genetic and Molecular Epidemiology Group Spanish National Cancer Research Centre (CNIO), CIBERONC Madrid Spain; ^67^ Department of Gastroenterology and Hepatology University Hospitals Leuven Leuven Belgium; ^68^ Digestive Cancers Europe (DiCE) Brussels Belgium; ^69^ Hepato Pancreato Biliary and Liver Transplant Surgery of the Department of Surgery Oncology and Gastroenterology (DiSCOG) Padova University Padova Italy; ^70^ Department of Anesthesiology, Creta Interclinic Hospital Hellenic Healthcare Group, Heraklion Crete Greece; ^71^ Oxford Cancer Centre Oxford University Hospitals NHS Foundation Trust Oxford UK; ^72^ Division of Interventional Radiology European Institute of Oncology, IRCCS Milan Italy; ^73^ Department of Medical Oncology Bank of Cyprus Oncology Centre Nicosia Cyprus; ^74^ European Association for Palliative Care Vilvoorde Belgium; ^75^ Department of Surgery TUM Universitätsklinikum Klinikum Rechts der Isar Technische Universität München Munich Germany; ^76^ German Cancer Consortium (DKTK), Partner Site Munich Germany; ^77^ Clinic for Digestive Surgery, University Clinical Centra of Serbia, Medical Faculty University of Belgrade Belgrade Serbia; ^78^ Hepato‐Pancreato‐Biliary Unit, Department of Surgery University Hospitals of Birmingham Birmingham UK; ^79^ Pancreatic Surgery Unit, Department of Engineering for Innovation Medicine (DIMI) University of Verona Verona Italy; ^80^ Department of HPB Surgery, AP‐HP, Hôpital Beaujon University of Paris Clichy France; ^81^ Arbeitskreis der Pankreatektomierten e.V. (AdP e.V.) Bonn Germany; ^82^ German Cancer Society Berlin Germany; ^83^ Department of Internal Medicine I University Hospital Ulm Ulm Germany; ^84^ Hepatobiliary Unit, Department of General and Visceral Surgery, Kepler University Hospital GmbH Johannes Kepler University Linz Austria; ^85^ Digestive Endoscopy Centre Hospitalier Universitaire de Nice Nice France; ^86^ Department of Pathology, Institute of Clinical Medicine University of Oslo Oslo Norway; ^87^ Department of Anaesthesiology, Intensive Care and Pain Therapy Saarland University Medical Centre and Saarland University Faculty of Medicine Homburg Germany; ^88^ Department of General, Visceral and Transplant Surgery Ludwig‐Maximilians‐University Munich Munich Germany; ^89^ Department of Medical Imaging Hôpitaux Paris Saint Joseph & Marie Lannelongue Paris France; ^90^ Department of Medical Oncology Institut de cancérologie de Lorraine, Vandoeuvre‐lès‐ Nancy France; ^91^ Inserm INSPIIRE Université de Lorraine Nancy France; ^92^ Department of Oncology Odense University Hospital Odense Denmark; ^93^ Department of Medical Oncology Gustave Roussy Villejuif France; ^94^ Faculté de Médecine Université Paris Saclay, Le Kremlin‐Bicêtre France; ^95^ Amsterdam UMC, Location Vrije Universiteit Department of Surgery Amsterdam the Netherlands; ^96^ Amsterdam UMC, Location Vrije Universiteit, Department of Medical Oncology Lab of Medical Oncology Amsterdam the Netherlands; ^97^ Amsterdam UMC, location University of Amsterdam Department Epidemiology and Data Science Amsterdam the Netherlands; ^98^ Amsterdam Public Health Research Institute, Methodology Program Amsterdam the Netherlands; ^99^ Department of Surgery, Section for Upper Abdominal Surgery Sahlgrenska University Hospital Gothenburg Sweden; ^100^ Department of Surgery at Institute of Clinical Sciences, Sahlgrenska Academy University of Gothenburg Gothenburg Sweden

**Keywords:** cancer treatment, guideline, multi‐societal, pancreatic cancer, PDAC

## Abstract

**Background:**

Pancreatic cancer remains one of the most lethal cancers despite extensive efforts and research conducted over the past decades. To effectuate groundbreaking improvements in pancreatic cancer treatment, interdisciplinary and international collaboration is essential. Evidence‐based guidelines, including state‐of‐the‐art evidence and expert opinion, are crucial to guide medical specialists, researchers, and patients, especially on issues where consensus is still lacking. This article describes the methodological protocol for the development of the European Multidisciplinary Evidence‐Based Guideline on Pancreatic Cancer. The guideline aims to identify current knowledge gaps on pancreatic cancer management, develop questions based on these knowledge gaps, and answer these questions with evidence‐based recommendations supplemented, when evidence is lacking, with expert advice for treatment and future research.

**Methods:**

This guideline development protocol is developed according to the Grading of Recommendations Assessment, Development, and Evaluation (GRADE) methodology. The process is structured into six stages: First, 13 theme‐based multidisciplinary working groups are established, comprising representatives from 30 European medical and patient societies. Second, these working groups identify the most relevant current knowledge gaps on pancreatic cancer within their theme and formulate key questions. Third, the available evidence to answer these key questions is obtained through systematic reviews and the certainty of evidence is assessed using the GRADE approach. Fourth, recommendations are developed based on the available evidence. Fifth, all participants reach consensus on the recommendations through a modified Delphi process. Sixth, the recommendations are discussed during an open conference, including an external validation committee.

**Discussion:**

This methodological protocol of the European Multidisciplinary Evidence‐Based Guideline on Pancreatic Cancer is designed to identify key knowledge gaps across 13 themes and formulate evidence‐based recommendations. This guideline initiative unites 30 European medical and patient societies for pancreatic cancer.

## Summary

- Methodological protocol of an international multi‐disciplinary guideline involving 30 European medical and patient societies.
- This protocol highlights the six stages of the guideline development process, adhering to the key principles of the GRADE methodology, and thereby aims to maximise transparency and methodological quality.
- With addressing knowledge gaps, this guideline will uncover areas with limited evidence. A priority rating for research needs, based on a survey, will be established, highlighting areas where future research is most warranted.

**Abbreviations**
AGREE‐II: Appraisal of Guidelines for Research & Evaluation II
CJ: Considered Judgement
E‐AHPBA: European‐African Hepato‐Pancreato‐Biliary Association
ESMO: European Society for Medical Oncology
ESSO: European Society of Surgical Oncology
EtD: Evidence to Decision
GRADE: Grading of Recommendations Assessment, Development, and Evaluation
ICMJE: International Committee of Medical Journal Editors
KQ: Key question(s)
OS: Overall survival
PICO: Population Intervention Control Outcome framework
PRISMA: Preferred Reporting Items for Systematic Reviews and Meta‐Analyses
QRG: Quick Reference Guide
RCT: Randomised Controlled Trial
SIGN 50: Scottish Intercollegiate Guidelines Network, publication no.50
SoF: Summary of Findings
UEG: United European Gastroenterology
WG: Working group(s)

## 1 Background

Pancreatic cancer is one of the most lethal malignancies, with a lifetime risk of 2% in high‐income countries [1, 2]. Notably, Western Europe has the highest lifetime risk of developing pancreatic cancer [2]. Despite decades of extensive research efforts, only moderate progress has been achieved in terms of overall survival (OS), with the most notable advancements for patients with resectable, borderline resectable and locally advanced disease who undergo surgery combined with systemic treatment [3, 4, 5, 6]. Herein, increasingly the anatomy, biologic (e.g. CA19.9), and conditional (i.e. performance status) ‘ABC’ criteria are taken into account rather than anatomy only [7]. The prognosis for metastatic pancreatic cancer remains dismal, underscoring the urgent need for innovative therapeutic strategies [8]. In addition to the biological challenges of pancreatic cancer, local differences in treatment contribute to differences in patient outcomes [9, 10, 11, 12, 13]. These variations reduce the overall quality of care and highlight the need for an internationally standardised, evidence‐based care pathway.

Recognising this need, various national and international societies and registries have been established to improve care for patients with pancreatic cancer through scientific and clinical collaboration [14, 15, 16, 17, 18, 19, 20]. Amidst the rapid pace of studies, international guidelines are crucial to achieve this improvement, as they provide up‐to‐date guidance and serve as a reference point for healthcare experts and patients, while ensuring consistency and clarity in decision‐making. Guidelines based on a thorough evaluation of the evidence combined with expert consensus are valuable to guide both medical specialists and patients through numerous issues where evidence‐based, standardised care pathways are still lacking. Furthermore, this approach can highlight topics which remain underrepresented in current scientific research, warranting the need for future research [21]. In addition to providing evidence‐based recommendations, guidelines ensure that advances in medical research are rapidly and effectively translated into clinical practice. Given the complexity of pancreatic cancer management and the critical importance of multimodal treatment approaches, as well as tailored supportive and palliative care concepts, guidelines must incorporate all relevant stakeholders and societies. Most current guidelines have focused on one specific aspect of pancreatic cancer or have originated from a single clinical discipline or country [22, 23, 24].

To address these limitations, the European‐African Hepato‐Pancreato‐Biliary Association (E‐AHPBA) and the European Society of Surgical Oncology (ESSO) initiated the development of the European Multidisciplinary Evidence‐Based Guideline on Pancreatic Cancer. This initiative seeks to integrate expertise from all relevant medical specialities along with patient partners, providing evidence‐based recommendations that focus specifically on knowledge gaps in the management of pancreatic cancer. This guideline aims to identify research priorities, promote innovation, and improve patient care by addressing these knowledge gaps. Here, we describe the methodological protocol for the development of the European Multidisciplinary Evidence‐Based Guideline on Pancreatic Cancer.

## 2 Methods

The European Multidisciplinary Guideline on Pancreatic Cancer was initiated in 2023 by a formal mandate from the councils of E‐AHPBA and ESSO, who invited two coordinators (MGB and ER) to oversee and guide the entire development process. Subsequently, two daily coordinators (LCL and MH) were appointed. The primary objective was to develop recommendations to address the most significant knowledge gaps in pancreatic cancer through an evidence‐based, systematic, interdisciplinary, and interprofessional approach at a European level. This project focuses on pancreatic ductal adenocarcinoma (PDAC) and intraductal papillary mucinous neoplasm (IPMN)‐associated PDAC. Consequently, the term “pancreatic cancer” is hereafter used to refer to this specific entity. The following section provides a detailed explanation of the guideline development process and the six distinct methodological stages that were followed.

### 2.1 Working Groups

The guideline development process is organised in 13 theme‐based working groups (WG), each focusing on a specific domain of pancreatic cancer management. Each WG included multidisciplinary representatives from the participating European societies (approximately 5–15 experts per group) together with 2–4 research leads. The working groups are responsible for identifying key questions within their theme, conducting systematic reviews and developing evidence‐based recommendations, all according to the predefined methodological frameworks.

### 2.2 Research Leads

PhD candidates, research fellows, and residents with expertise in pancreatic cancer management are invited as research leads for the 13 tWG. Research leads are responsible for the daily coordination of the WG and for conducting the systematic reviews under the supervision of the working group experts, methodologists and coordinators.

### 2.3 Patient Partners

All partners from patient societies participate in the guideline development process through a dedicated Patient Partners Group coordinated by the guideline coordinators. Patient partners are invited to join the working groups according to their interests and availability. As the systematic review process and technical meetings can be demanding, participation in the working groups is optional. A minimum of two meetings are held with the Patient Partners Group to review and discuss project progress and discuss results. Prior to the Delphi consensus procedure, draft recommendations are presented and discussed with the patient partners in lay language. This allows them to assess whether the recommendations align with patient perspectives and to provide suggestions for clarification or additional remarks. Their feedback is considered when finalising the recommendations, particularly with regard to patient values and preferences, and is considered in the development of the remarks accompanying the recommendations.

### 2.4 Methodologists

The entire guideline process is overseen by two experienced methodologists from Amsterdam UMC (ML and YV). Here, ML is a board member of the international Grading of Recommendations Assessment, Development, and Evaluation (GRADE) working group and co‐chair of the Dutch GRADE network.

### 2.5 Steering Committee

A Steering Committee is established to oversee feasibility and accuracy throughout the project. This committee consists of the guideline coordinators, representatives from E‐AHPBA and ESSO, one representative from each participating society, all research leads and methodologists. The committee reviews and approves outcomes at each stage before progressing to subsequent phases.

### 2.6 External Validation Committee

An independent committee of international pancreatic cancer experts will be established. These experts are not involved in the guideline development process and do not serve as representatives of the participating societies. They will independently review the draft guideline to assess its methodological quality, clinical relevance, and applicability, using the AGREE II instrument [25].

All individuals involved in the guideline process must disclose potential conflicts of interest (COI) prospectively to ensure transparency and integrity. COI declarations were collected at the start of the development process using the standardised ICMJE disclosure form, regardless of the individual's role in the project. The disclosures were collected and reviewed by the guideline coordinators. Authorship of this protocol and future publications arising from the guideline project follows the ICMJE authorship criteria.

## 3 Framework: Six Stages

**Figure 1:**
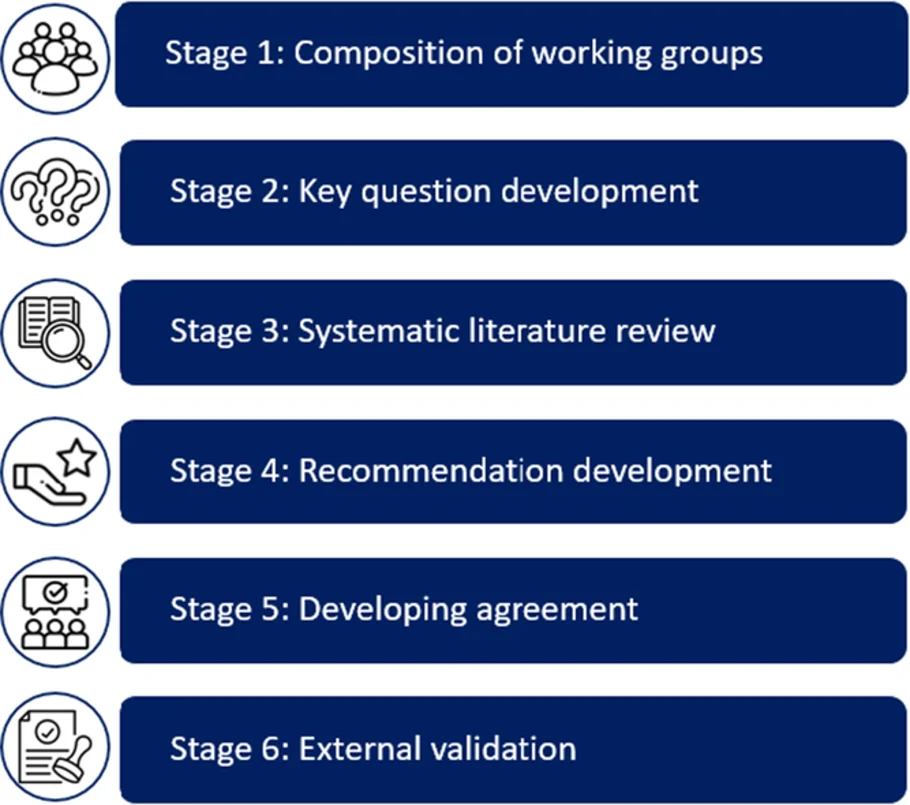
Flow chart of guideline development stages.

The guideline development process follows six clearly defined stages, adhering to the key principles of the GRADE methodology [26]. To support structured and transparent documentation of the recommendation development process across the multiple working groups involved in the project, Considered Judgement templates adapted from the SIGN framework were used to record the rationale underlying the recommendations [27, 28]. In addition, the development process was designed in accordance with the framework for high‐quality clinical guideline development proposed by the United European Gastroenterology (UEG) [29]. The development of this protocol and the guideline was conducted in alignment with the domains of the AGREE II instrument to ensure methodological rigour and transparency. An overview of all project stages is provided in Figure 1.

To ensure a high standard and consistent execution across all stages, a detailed methodology manual is developed and provided as a reference for all guideline members. Where applicable, this guideline also incorporates and references existing high‐quality guidelines, fostering alignment and avoiding redundancy.

Starting in 2023, the four coordinators (MGB, ER, LCL, MH) invited European medical and patient societies in the field of pancreatic cancer. Prior to inviting these societies, the overall scope of the guideline and 13 predefined thematic areas of pancreatic cancer management had been established to guide invitations and composition of the WG. In February 2024, 30 European societies and organisations acknowledged the need for a multidisciplinary approach to improve international management of pancreatic cancer and provided their official endorsement (Table 1). Each participating society and organisation was requested to provide two to three representatives, ensuring diverse and balanced input from across Europe. ‘Observer‐members’ from other societies can be invited to ensure accurate referencing to their respective societal guidelines, where applicable, such as the European Society for Medical Oncology (ESMO) and their Clinical Practice Guideline for pancreatic cancer [30]. ESMO has a longstanding policy of not participating in multidisciplinary multi‐societal guidelines, but provides three “observers” to ensure that the ESMO guideline is optimally referenced and integrated in the current guideline. In addition to the societies, 42 research leads (i.e. PhD candidates, research fellows and clinical trial experts) with expertise in pancreatic cancer management from across Europe were invited.

**Table 1:** Overview of Participating Societies.

	Society
1 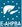	European‐African Hepato‐Pancreato‐Biliary Association (E‐AHPBA)
2 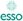	European Society of Surgical Oncology (ESSO)
3 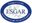	European Society of Gastrointestinal and Abdominal Radiology (ESGAR)
4 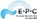	European Pancreatic Club (EPC)
5 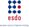	European society of digestive oncology (ESDO)
6 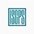	International study group of pancreatic surgery (ISGPS)
7 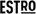	European Society for Radiotherapy and Oncology (ESTRO)
8 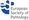	European Society of Pathology (ESP)
9 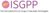	International Study Group of Pancreatic Pathologists (ISGPP)
10 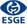	European Society of Gastrointestinal Endoscopy (ESGE)
11 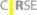	Cardiovascular and interventional Radiological Society of Europe (CIRSE)
12 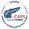	European Consortium on Advanced Pancreatic Surgery (E‐CAPS)
13 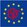	European Society of Regional Anaesthesia and pain therapy (ESRA)
14 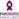	Pancreatic Cancer Europe (PCE)
15 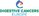	Digestive Cancers Europe (DiCE)
16 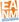	European association of Nuclear Medicine (EANM)
17 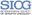	International Society of Geriatric Oncology (SIOG)
18 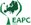	European Association for Palliative Care (EAPC)
19 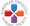	European Oncology Nursing Society (EONS)
20 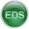	European Digestive Surgery (EDS)
21 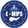	European Consortium on Minimally Invasive Pancreatic Surgery (E‐MIPS)
22 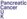	Pancreatic Cancer UK (PCUK)
23 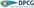	Dutch Pancreatic Cancer Group (DPCG)
24 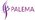	Cancer Society of PALEMA (PALEMA)
25 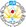	Arbeitskreis der Pankreatekomierten (AdP)
26 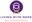	Living with Hope (LWH)
27 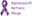	Pankreaskreft Nettverk Norge (PKNN)
28 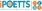	International Prehabilitation Society (iPOETTS)
29 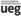	United European Gastroenterology (UEG)
30 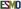	European Society for Medical Oncology (ESMO)**

** Guideline observer members.

### 3.1 Stage 1: Composition of Working Groups

In stage 1, to systematically address knowledge gaps across the entire patient journey of pancreatic cancer, 13 theme‐based WG are established (Table 2). The WG address all aspects of patient care. These groups serve as the primary units wherein the guideline development process is conducted. Each representative from the participating societies is invited to join the WG most aligned with their expertise and societal focus. This approach ensures that the WG includes experts from diverse healthcare backgrounds and organisations, minimising the risk of bias that could arise from uniform agendas within a single society. Importantly, representatives are allowed to join multiple WGs.

**Table 2:** Working Group Themes.

WG	Topic
1	Early detection, imaging at diagnosis and staging
2	Biliary drainage/biopsy
3	Neoadjuvant and induction therapy
4	Preoperative counselling and prehabilitation
5	Surgery
6	Postoperative in‐hospital management
7	External tumour‐directed percutaneous approaches
8	Adjuvant treatment
9	Palliative tumour‐directed systemic treatment
10	Medical treatment
11	Palliative and supportive care
12	Pathology
13	Follow‐up

Each researcher is assigned to one of the WGs based on their preferences, resulting in two to four researchers per WG. One primary research lead is appointed for each WG to coordinate and serve as the main point of contact for the entire group and the coordinators.

### 3.2 Stage 2: Key Question Development

In stage 2, the most relevant knowledge gaps are identified. This stage lays the foundation for defining three key questions (KQ) per theme‐based WG. The relevance of the KQ directly affects the quality and clinical applicability of the final recommendations. The 13 WG are asked to prioritise clinical relevance over the expected availability of evidence, with an emphasis on addressing key knowledge gaps. By doing so, remaining evidence gaps in the final stage can still be highlighted, guiding future research priorities. The KQ are formulated according to the Patient, Intervention, Comparison, Outcome (PICO) framework to ensure the feasibility of the systematic reviews. WG are allowed to propose up to three KQ, ensuring a realistic scope that allows in‐depth investigation while maintaining a concise and practical guideline for clinical use. With 13 WG, a maximum of 39 KQ will be developed. Once formulated, the Steering Committee reviews and, if necessary, refines and prioritises the final set of the KQ.

### 3.3 Stage 3: Systematic Literature Review

In stage 3, systematic literature reviews are performed for each KQ following the GRADE methodology. This ensures that the evidence base for each recommendation is reliable, well appraised, and synthesised to support the recommendations [31]. While evidence‐based recommendations are prioritised to minimise bias, it is acknowledged that addressing knowledge gaps will, almost by definition, uncover areas with limited evidence.

The literature search is managed by the research leads under the guidance of the methodologists and librarians. One librarian with extensive experience in systematic literature searches supported the development of all search strategies. Given the large scale of the guideline project, additional academic librarians with comparable expertise will assist in this process. A systematic literature search for each KQ is conducted using MEDLINE (via PubMed) and EMBASE, with predefined eligibility criteria. A core set of eligibility criteria was provided as a common methodological starting point for all working groups to ensure methodological consistency and feasibility across the large number of systematic reviews conducted within this guideline initiative. These criteria included general parameters such as language restrictions, publication year limits (studies published from 2000 onwards), and study design considerations. Specifically, studies were limited to those published in English, and eligible study designs included randomised controlled trials, observational studies, and relevant systematic reviews or guidelines addressing the key question. Case reports and conference abstracts were excluded due to methodological limitations, and paediatric studies were excluded as this guideline focuses on adult pancreatic cancer management. Minor adjustments to these criteria (e.g. publication year range or study design restrictions) were permitted when justified by the specific key questions, provided that the predefined PICO framework and scope of the questions remained unchanged. Any deviation had to be discussed within the working group and documented. The systematic review process and study selection are reported in accordance with the Preferred Reporting Items for Systematic Reviews and Meta‐Analyses (PRISMA) 2020 reporting guidelines, including presentation of the study selection process in a PRISMA flow diagram [32]. Screening is performed in two stages: title and abstract screening, followed by full‐text screening of potentially eligible articles. Screening of the search output for each KQ is conducted by one or two research leads, with methodological oversight from the coordinators and WG experts in case of uncertainties regarding article inclusion. Included studies are summarised in evidence tables for each included study. For the critical appraisal, various methodological tools and checklists are applied depending on the study type [32, 33, 34, 35]. When existing systematic reviews were included, the methodological quality was assessed using the AMSTAR‐2 framework [36]. Risk‐of‐bias assessments and interpretation of the evidence are discussed within the working groups to ensure methodological consistency. After data extraction, Summary of Findings (SoF) tables are created by the research leads for all predefined outcomes per KQ [37]. These provide key data on each outcome, if applicable, the absolute and relative effect, the volume of available evidence, and the certainty of the evidence in accordance to the GRADE methodology [38]. Given the probable variability of KQ and outcomes across WG, the types of studies and results may differ, resulting in SoF tables tailored to the specific context of each KQ. If applicable, a meta‐analysis is performed. If studies reported meta‐analyses, these were either replicated or updated in accordance with the current state‐of‐the‐art in meta‐analytic modelling. Random‐effects meta‐analyses for ‘generic’ endpoints (e.g. hazard ratios) were performed using a random‐effects model with a restricted maximum likelihood estimator and the modified Hartung‐Knapp/Sidik‐Jonkman variance correction [39]. If necessary, log‐transformation was used prior to pooling, after which summary estimates were back‐transformed to their original scale. Diagnostic meta‐analyses were performed using a bivariate generalised linear mixed model approach [40, 41] to estimate the summary sensitivity and specificity, assuming the use of a common cut‐off point. Meta‐analyses of (univariate) binary endpoints and odds ratios were performed using generalised linear mixed models [42].

The GRADE methodology is applied to assess the overall certainty of evidence for each specific outcome. To reach a final judgement on the quality of evidence, the GRADE methodology assesses different factors including the risk of bias, inconsistency, indirectness, imprecision, publication bias, large effect, plausible confounding and the dose‐response gradient [38]. The results are categorised into one of four GRADE levels of certainty (high, moderate, low, or very low, see Table 3) [43]. To support transparent documentation of this assessment, part (A) of the Considered Judgement form is used to summarise the GRADE domains considered in the evaluation [25, 26, 27]. As part of the European Multidisciplinary Guideline on Pancreatic Cancer, systematic reviews conducted during this stage can be published as secondary papers. To ensure methodological rigour and transparency, literature screening for systematic reviews that are aimed for secondary publication is always conducted by two independent reviewers. Furthermore, prospective registration of each systematic review for secondary publication in the PROSPERO registry is required, aligning with international standards for systematic review protocols.

**Table 3:** GRADE Levels of the Certainty of Evidence (43).

Quality level	Definition
High	The true effect lies close to that of the estimated effect.
Moderate	The true effect is likely to be close to the estimated effect, but there is a possibility that it is substantially different
Low	The true effect might be substantially different from the estimated effect
Very low	The true effect is likely to be substantially different from the estimated effect

### 3.4 Stage 4: Recommendation Development

In Stage 4, evidence‐based recommendations for each KQ are developed based on the systematic reviews and evidence assessments conducted in Stage 3. Recommendation development follows the principles of the GRADE methodology, considering the balance between desirable and undesirable effects, patient values and preferences, and feasibility.

To support structured documentation of the working groups’ deliberations when moving from evidence to recommendations, part B of the Considered Judgement form was used to record the rationale underlying the recommendations. These templates capture domains similar to those considered in the GRADE Evidence to Decision (EtD) framework, including the balance of benefits and harms, patient impact, and feasibility [26, 27, 44].

Together with the experts in each WG, the available evidence is evaluated to determine whether a ‘strong’ or ‘conditional’ recommendation should be formulated:

– ‘Strong Recommendations’ are made when there is high confidence that desirable effects outweigh the undesirable effects, taking into account the certainty of the evidence, the balance of benefits and harms, patient values and feasibility considerations [45].
– ‘Conditional Recommendations’ are made when there is uncertainty regarding the balance between desirable and undesirable effects, the certainty of the evidence is low or very low, or when patient preferences or implementation may vary across settings [45].

When evidence is limited or absent, expert opinion may be incorporated to support clinical guidance. In such cases, the limited evidence base is explicitly acknowledged, and expert input is clearly labelled as expert opinion to maintain a clear distinction between evidence‐based and consensus‐based components of the recommendations. Expert opinion may be systematically collected, for example, through structured surveys among the representatives, and used as additional input in the formulation of recommendations or remarks [45]. Expert opinion is not mandatory and is only incorporated when sufficient agreement among the experts is achieved.

### 3.5 Stage 5: Developing Agreement

In stage 5, consensus is reached among all guideline members on the developed recommendations through an online survey system. Given the large multidisciplinary and multinational structure of the guideline development group, a modified Delphi procedure is used to ensure structured consensus across the participating experts. The Delphi process ensures anonymous voting and systematic feedback from all participants, in multiple rounds [46]. A consensus threshold of ≥ 75% agreement is required for a recommendation to proceed to the final consensus meetings. For recommendations failing to meet this threshold in the first Delphi round, re‐discussion and re‐formulation are performed within the respective WG, implementing the given feedback, and documenting the rationale for all modifications made. Hereafter, with the revised recommendations, a second Delphi round will be conducted, following the same format as the first Delphi round. If the threshold is not met again, these recommendations will be given a final opportunity for correction, followed by a last third Delphi round. If a recommendation already meets the threshold in the first Delphi round and several respondents give similar comments that could improve the phrasing and thus agreement, one additional Delphi round is allowed. However, the updated recommendation will only be used if (i) it meets the ≥ 75% agreement threshold and (ii) in a direct head‐to‐head question, the updated recommendation receives the majority of votes. Recommendations for which consensus remains unattainable by the final third round will not proceed to the final consensus meeting and will not be included in the guideline. However, these recommendations will be listed as a supplement in the guideline publication to ensure transparency. The Delphi procedure will be reported in accordance with the principles of the ACCORD guideline [47]. Next, a priority rating of research needs based on the identified knowledge gaps and recommendations will be established during a single voting round. Finally, each WG is asked to provide ‘best practice statements’ regarding the management of pancreatic cancer within their domain. This will not include knowledge gaps, as these are addressed in the guideline itself. These ‘best practice statements’ will be listed as a separate section of the guideline.

### 3.6 Stage 6: External Validation

In stage 6, all WG present their recommendations in an online meeting of all those involved with the guideline. The purpose of this meeting is to finalise the wording and ensure uniformity in phrasing across all recommendations. Considerable changes to the content of the recommendations are not permitted at this stage, as these should have been resolved during the Delphi rounds. At this stage, the External Validation Committee will also review the recommendations and the complete guideline using the AGREE II‐Global Rating Scale tool to ensure adequate methodological and guideline quality.

Hereafter, an open discussion regarding the recommendations will take place at a 1‐day public conference, held during the 2025 E‐AHPBA Congress in Dublin. The conference will allow previously uninvolved medical specialists and members of the public to attend and comment on the recommendations and specific aspects of the guideline. These comments will be added to the final guideline report. During this open meeting, the external validation committee will also attend and review the process. The methodologists advised to not establish a voting procedure during the final meeting, as during the open access meeting, COI cannot be prevented; and in the guideline development group, all relevant stakeholders were represented, who, during the multiple Delphi rounds, were able to provide feedback with an equal vote.

## 4 Dissemination Policy

The guideline will be published in a peer‐reviewed medical journal following all parties’ final approval, adhering to the International Committee of Medical Journal Editors (ICMJE) authorship guidelines. To enhance its reach, long summaries that include key recommendations will be featured in relevant society journals, and secondary manuscripts for individual systematic reviews conducted during the guideline development are allowed to be published. A Quick Reference Guide (QRG) will accompany the main publication, offering a concise summary of the key recommendations and clinical pathways for ease of use in practice. The guideline will also be submitted for inclusion in the UEG GI Guidelines App, enabling convenient digital access. Patient versions of the guideline will be developed to facilitate communication between healthcare providers and patients, ensuring accessibility and understanding to all patients.

## 5 Guideline Updates

To maintain clinical relevance, the guideline will be monitored for emerging evidence relevant to the addressed key questions. Rather than implementing fixed full guideline revisions at predefined intervals, updates will be performed in a modular manner when substantial new evidence or landmark studies become available within specific thematic areas. When such updates are required, relevant evidence will be assessed using the same methodological framework described in this protocol. This approach allows timely and focused updates while maintaining methodological consistency and transparency [19].

## 6 Discussion

The European Multidisciplinary Evidence‐Based Guideline on Pancreatic Cancer aims to address current knowledge gaps by providing evidence‐based recommendations for the management of pancreatic cancer. The present article describes the methodological protocol underlying the development of this guideline. The guideline is intended to identify and prioritise key areas for future research based on the identified knowledge gaps. This methodological protocol aims to maximise transparency and uphold high methodological standards. While not yet as standardised in practice as guideline assessment tools such as AGREE II [36], the publication of guideline protocols is increasingly recognised as a hallmark of methodological quality [48, 49, 50, 51].

A distinguishing feature of the current guideline is its multidisciplinary and interprofessional composition, which fosters collaboration among all relevant stakeholders, including patient partners. Integrating the systematic development of KQ as a distinct stage in the guideline process (stage 2) ensures objectivity by concentrating on the most current and relevant knowledge gaps, rather than relying on predefined agendas that may introduce bias. Previous guidelines typically focus either on specific aspects of the patient pathway or predetermine the topics to be addressed before the development process begins [22, 23, 24].

Several considerations related to this protocol should be acknowledged. First, limitations in the available evidence are anticipated, as the resulting guideline specifically focuses on knowledge gaps in the management of pancreatic cancer. For some key questions, high‐quality evidence may therefore be limited. Expert opinion may be incorporated where necessary to support recommendations or accompanying remarks, with a clear distinction maintained between the evidence‐based and expert‐informed guidance. Second, given the large number of key questions addressed across multiple working groups, duplicate screening is not feasible for every systematic review. To mitigate potential bias, the review process is conducted under methodological supervision, and study inclusion and risk‐of‐bias assessments are discussed within the multidisciplinary working groups.

The overarching goal of the guideline is to create inclusive, evidence‐based guidance for key knowledge gaps in pancreatic cancer care, and highlight areas where future research remains warranted, while fostering multidisciplinary collaboration among European societies. The publication of this protocol highlights the commitment to transparency and methodological quality, supporting the reliability and confidence of the recommendations from this guideline.

## Author Contributions

Study concept and design: Laura C. Leeuwenburgh, Magdalena Holze, Miranda Langendam, Yasaman Vali, Elena B. Rangelova, Marc G. Besselink. Analysis and interpretation of data: Laura C. Leeuwenburgh, Magdalena Holze, Dena Akhoundzadeh, Adnan Alsourani, Ricardo Alvarez Jimenez, Paul C. M. Andel, Charlotte Baggerman van Houweninge, Alberto Balduzzi, Caro L. Bruna, Fabio Casciani, Alice Cattelani, Aniek E. van Diepen, Heleen Driessens, Daphne H. M. Droogh, Jeska A. Fritzsche, Eline van Gansewinkel, Luana Genova, Anne M. Gehrels, Gonzalo Gómez Dueñas, Riccardo Guastella, Lis S. M. Hoeijmakers, Tijs J. Hoogteijling, Ammar A. Javed, Berk Kaan Aktas, Benedict Kinny‐Köster, Amber M. Lamoré, K. Seng Liem, Gabriella Lionetto, Lorenzo Lo Faro, Lisanne Lutter, Manuela Mastronardi, Carlo H. Maurer, Roberto M. Montorsi, Julian Musa, Ingmar F. Rompen, Jacobien C. M. Scheepens, Dana Sochorová, Thomas F. Stoop, Nuray Tezcan, Bas A. Uijterwijk, Roberta Vella, Federica Vernuccio, Rogier P. Voermans, Mohammad Abu Hilal, Volkan Adsay, Hana Algül, Valentina Ambrosini, Livia Archibugi, Eva Backman, Sorin T. Barbu, Ugo Boggi, Charlotte Borge‐Andersen, Stefan Kobbelgaard Burgdorf, Alice Clarkson, Eric van Cutsem, Pieter‐Jan Cuyle, Lois A. Daamen, Peter De Rosa, Marcel den Dulk, Celia Diez de los Rios, Amanda Drury, Irene Esposito, Simon M. Everett, Arantza Farina Sarasqueta, Isabella Frigerio, Bas Groot Koerkamp, Thilo Hackert, Florence Huguet, Nikolaos Kartalis, Anne Letsch, Pex Langenberg, Denny Levett, Gabor Liposits, Núria Malats, Hannah van Malenstein, Giuseppe Malleo, Zorana Maravic, Giovanni Marchegiani, Ana Martins, Martijn R. Meijerink, Christoph W. Michalski, J. Sven D. Mieog, Eleni Moka, Somnath Mukherjee, Cristina Nanni, Franco Orsi, Demetris Papamichael, Stefano Partelli, Cathy Payne, Ilaria Pergolini, Dejan Radenkovic, Keith J. Roberts, Roberto Salvia, Alain Sauvanet, Marta Scorsetti, Matthias Seebo, Thomas Seufferlein, Stefan Stättner, Geoffroy Vanbiervliet, Caroline S. Verbeke, Thomas Volk, Jens Werner, Johanna W. Wilmink, Marc Zins, Thierry Conroy, Per Pfeiffer, Michel Ducreux, Mahsoem Ali, Miranda Langendam, Yasaman Vali, Elena B. Rangelova, Marc G. Besselink. Drafting of the manuscript: Laura C. Leeuwenburgh, Magdalena Holze, Miranda Langendam, Yasaman Vali, Mahsoem Ali, Elena B. Rangelova, Marc G. Besselink. Critical revision of the manuscript for important intellectual content: Laura C. Leeuwenburgh, Magdalena Holze, Dena Akhoundzadeh, Adnan Alsourani, Ricardo Alvarez Jimenez, Paul C. M. Andel, Charlotte Baggerman van Houweninge, Alberto Balduzzi, Caro L. Bruna, Fabio Casciani, Alice Cattelani, Aniek E. van Diepen, Heleen Driessens, Daphne H. M. Droogh, Jeska A. Fritzsche, Eline van Gansewinkel, Luana Genova, Anne M. Gehrels, Gonzalo Gómez Dueñas, Riccardo Guastella, Tijs J. Hoogteijling, Ammar A. Javed, Berk Kaan Aktas, Benedict Kinny‐Köster, Amber M. Lamoré, K. Seng Liem, Gabriella Lionetto, Lorenzo Lo Faro, Lisanne Lutter, Manuela Mastronardi, Carlo H. Maurer, Roberto M. Montorsi, Julian Musa, Ingmar F. Rompen, Jacobien C. M. Scheepens, Dana Sochorová, Thomas F. Stoop, Nuray Tezcan, Bas A. Uijterwijk, Roberta Vella, Federica Vernuccio, Rogier P. Voermans, Mohammad Abu Hilal, Volkan Adsay, Hana Algül, Valentina Ambrosini, Livia Archibugi, Eva Backman, Sorin T. Barbu, Ugo Boggi, Charlotte Borge‐Andersen, Stefan Kobbelgaard Burgdorf, Alice Clarkson, Eric van Cutsem, Pieter‐Jan Cuyle, Lois A. Daamen, Peter De Rosa, Marcel den Dulk, Celia Diez de los Rios, Amanda Drury, Irene Esposito, Simon M. Everett, Arantza Farina Sarasqueta, Isabella Frigerio, Bas Groot Koerkamp, Thilo Hackert, Florence Huguet, Nikolaos Kartalis, Anne Letsch, Pex Langenberg, Denny Levett, Gabor Liposits, Núria Malats, Hannah van Malenstein, Giuseppe Malleo, Zorana Maravic, Giovanni Marchegiani, Ana Martins, Martijn R. Meijerink, Christoph W. Michalski, J. Sven D. Mieog, Eleni Moka, Somnath Mukherjee, Cristina Nanni, Franco Orsi, Demetris Papamichael, Stefano Partelli, Cathy Payne, Ilaria Pergolini, Dejan Radenkovic, Keith J. Roberts, Roberto Salvia, Alain Sauvanet, Marta Scorsetti, Matthias Seebo, Thomas Seufferlein, Stefan Stättner, Geoffroy Vanbiervliet, Caroline S. Verbeke, Thomas Volk, Jens Werner. Johanna W. Wilmink, Marc Zins, Thierry Conroy, Per Pfeiffer, Michel Ducreux, Mahsoem Ali, Miranda Langendam, Yasaman Vali, Elena B. Rangelova, Marc G. Besselink. Statistical analysis: Laura C. Leeuwenburgh, Magdalena Holze, Dena Akhoundzadeh, Adnan Alsourani, Ricardo Alvarez Jimenez, Paul C. M. Andel, Charlotte Baggerman van Houweninge, Alberto Balduzzi, Caro L. Bruna, Fabio Casciani, Alice Cattelani, Aniek E. van Diepen, Heleen Driessens, Daphne H. M. Droogh, Jeska A. Fritzsche, Eline van Gansewinkel, Luana Genova, Anne M. Gehrels, Gonzalo Gómez Dueñas, Riccardo Guastella, Lis S. M. Hoeijmakers, Tijs J. Hoogteijling, Ammar A. Javed, Berk Kaan Aktas, Benedict Kinny‐Köster, Amber M. Lamoré, K. Seng Liem, Gabriella Lionetto, Lorenzo Lo Faro, Lisanne Lutter, Manuela Mastronardi, Carlo H. Maurer, Roberto M. Montorsi, Julian Musa, Ingmar F. Rompen, Jacobien C. M. Scheepens, Dana Sochorová, Thomas F. Stoop, Nuray Tezcan, Bas A. Uijterwijk, Roberta Vella, Federica Vernuccio, Rogier P. Voermans, Mahsoem Ali. Obtained funding: Not applicable. Study supervision: Laura C. Leeuwenburgh, Magdalena Holze, Miranda Langendam, Yasaman Vali, Elena B. Rangelova, Marc G. Besselink. All the authors have made substantial contributions to the design of the work, the analysis and interpretation of the data, and the drafting of the work or its critical revision.

## Funding

The authors have nothing to report.

## Ethics Statement

The European Multidisciplinary Evidence‐Based Guideline on Pancreatic Cancer will be conducted in accordance with the current version of the Declaration of Helsinki. No artificial intelligence tools will be used during the guideline development process.

## Conflicts of Interest

All authors completed an ICMJE disclosure form. RA‐J declares payment or honoraria from Viatris, paid to Amsterdam UMC, for a webinar on pain in chronic pancreatitis; support for attending meetings from Medtronic and Boston Scientific; and a patent or patent‐related interest involving Novac air, stated to be outside the scope of this project. TV declares grants from Sedana Medical, Ratiopharm, Saarland, Pfizer, InfectoPharm, and CytoSorbents Europe GmbH, paid to their institution; lecture fees from Pajunk and CSL Behring, paid personally; and a past‐president role with the European Society of Regional Anaesthesia and Pain Therapy. PDR declares travel support from the PRECEDE Consortium to attend the PRECEDE Consortium Annual Conference; full‐time employment as Senior Policy Manager at Pancreatic Cancer UK; and notes that Pancreatic Cancer UK occasionally receives very small donations from the pharmaceutical industry, which are not related to this project or his role within the organisation. AAJ declares grants, paid to their institution, from the Herb Kosten Foundation, Witches of St Andrews, the Nikki Mitchell Foundation and Pancreas Club, and the PRIA Foundation for research on pancreatic cancer biomarkers and circulating tumour cell culturing; personal payment or honoraria from the University of Oklahoma for the 2023 Symposium on Pancreatic Cancer Cachexia; and receipt by their institution of a Rare cells ISET device for research on circulating tumour cell biology. TFS declares grants from the Dutch Cancer Society (KWF) and Delta plan Alvleesklierkanker for the Dutch PREOPANC‐4 trial on multidisciplinary management of locally advanced pancreatic cancer, paid to their institution; and personal travel support from Cancer Center Amsterdam and the Cultuur fonds. FV declares honoraria from Bracco Suisse SA; support for attending meetings or travel from Bracco Imaging, Guerbet, and GE HealthCare; and membership of the ESGAR Membership Committee and Young ESGAR Coordinating Team, the EuSoMII Scientific Committee, the RSNA Regional Committee for Europe, the scientific editorial board of Insights into Imaging, and the editorial board of AJR. RPV declares a research grant from Prion Medical; consulting fees from Boston Scientific and Cook Medical; and payment or honoraria for lectures or educational events from Boston Scientific and Viatris. VAd declares leadership roles as Head of the Pathology Department and Chair of the Department of Surgical Sciences, Koç University, Turkey; past President of the Pancreatobiliary Pathology Society; and past President of the United States and Canadian Academy of Pathology. VAmb declares personal payment or honoraria from EANM, ESMIT, ESMO, Elma Academy, and AAA; travel support from Tema to attend the AIMN2024 conference, paid to their institution as an educational grant; participation on the ENETS advisory board and as lead of the theranostic task force, ESMO faculty for neuroendocrine tumours, and the EAN Moncology and theranostic committee; and personal honoraria from Cineca for developing questions for Italian national examinations. LA declares a research grant from AIRC (Associazione Italiana Ricerca sul Cancro) for project IG26201; payment or honoraria from Boston Scientific and Endoscopy on Air for lectures; and an unpaid role as UEG Quality of Care EPC representative and leader of the UEG project on pancreatic cancer quality indicators. SKB declares support for attending meetings from Intuitive for IHPBA 2022 and Medtronic for IHPBA 2024. AC declares that Pancreatic Cancer UK occasionally receives very small donations from the pharmaceutical industry, which are not related to this project or her role within the organisation. PJC declares consulting fees from BMS, Servier, and AstraZeneca; payment or honoraria from Ipsen; support for attending meetings or travel from Ipsen and Servier; and advisory board participation for BMS, Servier, and AstraZeneca. CDRdlS declares participation in EONS research and educational projects on hepatocellular cancer and breast cancer, includingABC4nurses and RCC4nurses, supported by Pfizer, and the HCC PROMs project, supported by Ipsen. AD declares consulting fees from the European Commission and Sharing Progress in Cancer Care; participation on the European Cancer Mission Board and the European Commission Initiative for Colorectal Cancer Working Group; and a board role with the European Oncology Nursing Society. NK declares consulting fees from Bayer, paid to their institution; payment or honoraria for lectures, presentations, speakers' bureaus, manuscript writing, or educational events from Bayer, Ascelia Pharma, and Guerbet, paid to their institution; participation on an advisory board for Ascelia Pharma, paid totheir institution; and unpaid membership of the executive committees of the European Society for Gastrointestinal and Abdominal Radiology and the Swedish Society for Gastrointestinal and Abdominal Radiology. HvM declares consulting fees from Boston Scientific. GM declares consulting fees from Oncosil Medical for the TRIPP_FFX randomised trial, and participation on an advisory board and safety monitoring board for Oncosil Medical for the TRIPP_FFX randomised trial. EM declares fees from Menarini for lectures or presentations; support from the European Society of Regional Anaesthesia and Pain Therapy for attending meetings; and leadership roles as President of the European Society of Regional Anaesthesia and Pain Therapy, Vice Chair of ESRA Hellas, and Ex‐Officio member of the board of the ESRA European Diploma of Regional Anaesthesia. SM declares grants from Oxford University and Genesis Care UK, paid to their institution, for the EMERALD trial, for which SM is chief investigator. FO declares payment or honoraria from Medtronic for a webinar on microwave ablation and from IGEA for a lecture on electrochemotherapy; and support from IGEA for attending the ESIR conference in Edinburgh. RS declares a grant from PNRR Heal Italia 2021,paid to their institution; a patent for a pancreas‐specific stapler cartridge; leadership roles as Editor‐in‐Chief of Digestive Surgery and board member of the Fondazione Italiana per la Ricerca sulle Malattie del Pancreas; receipt of equipment, materials, drugs, medical writing, gifts, or other services from ESAOTE S.p.A., paid to their institution; and research collaborations with MoviS.p.A., BeiGene, Edward Lifesciences S.p.A., AbbVie, and Eli Lilly and Company,paid to their institution. AS declares membership of the editorial board of Journal of Visceral Surgery, Elsevier. SS declares payment or honoraria from AstraZeneca for advisory board participation and presentations; from Roche,Bristol Myers Squibb, Takeda, Incyte, and Mölnlycke for presentations; and receipt of an unrestricted grant from ITM Pharma for the European Society of Surgical Oncology. GV declares personal honoraria for lectures from Norgine, Tillotts, Takeda, Fujifilm, and Pentax; and personal honoraria for advisoryboard participation from Ambu. JWW declares grants from MSD, Servier, and Nordic, paid to their institution; consulting fees from MSD, AstraZeneca, and Servier, paid to their institution; and participation on an advisory board for MSD, paid to their institution. MZ declares support for attending executive committee meetings and programme planning committees of the European Society for Gastrointestinal and Abdominal Radiology, and a leadership role as currentpresident of the European Society for Gastrointestinal and Abdominal Radiology.MGB declares research grants from Intuitive Surgical, Oncosil, Ethicon, and Medtronic, paid to their institution. DL declares grants from NIHR BRC Southampton, an unrestricted educational grant from BD Medical for aperioperative risk study, and an NIHR Programme grant for prehabilitation in cardiac surgery; and leadership roles as Director of the Centre for Perioperative Care at the Royal College of Anaesthetists, President of the International Prehabilitation Society, NHSE system perioperative medicine lead for Hampshire and Isle of Wight, Chair of the national perioperative CPET course, Perioperative Medicine Clinical Lead at University Hospital Southampton, Theme Lead for Perioperative and Critical Care at NIHR Southampton BRC, board member of Evidence Based Perioperative Medicine Conferences, Chair of the World Prehabilitation Conference 2019/2023, Chair of perioperative CPET guidelines, and Director of Prehab for Life. All authors declare no other competing interests.

## Supporting information

Supporting File

## Data Availability

Data sharing not applicable to this article as no datasets were generated or analysed during the current study.
