# Supplementary material for: The European Multidisciplinary Evidence‐Based Guideline on Pancreatic Cancer: Methodological Protocol
Source: Clin Public Health Guidel. 2026 Jul 13;3(3):e70074. doi: 10.1002/gin2.70074 (PMC13563389; doi:10.1002/gin2.70074)
Supplement: Supplementary file 1 [file GIN2-3-e70074-s001.docx]

**Table 1. Overview of participating societies**

|  | Society |
| --- | --- |
| 1 | European-African Hepato-Pancreato-Biliary Association (E-AHPBA) |
| 2 | European Society of Surgical Oncology (ESSO) |
| 3 | European Society of Gastrointestinal and Abdominal Radiology (ESGAR) |
| 4 | European Pancreatic Club (EPC) |
| 5 | European society of digestive oncology (ESDO) |
| 6 | International study group of pancreatic surgery (ISGPS) |
| 7 | European Society for Radiotherapy and Oncology (ESTRO) |
| 8 | European Society of Pathology (ESP) |
| 9 | International Study Group of Pancreatic Pathologists (ISGPP) |
| 10 | European Society of Gastrointestinal Endoscopy (ESGE) |
| 11 | Cardiovascular and interventional Radiological Society of Europe (CIRSE) |
| 12 | European Consortium on Advanced Pancreatic Surgery (E-CAPS) |
| 13 | European Society of Regional Anaesthesia and pain therapy (ESRA) |
| 14 | Pancreatic Cancer Europe (PCE) |
| 15 | Digestive Cancers Europe (DiCE) |
| 16 | European association of Nuclear Medicine (EANM) |
| 17 | International Society of Geriatric Oncology (SIOG) |
| 18 | European Association for Palliative Care (EAPC) |
| 19 | European Oncology Nursing Society (EONS) |
| 20 | European Digestive Surgery (EDS) |
| 21 | European Consortium on Minimally Invasive Pancreatic Surgery (E-MIPS) |
| 22 | Pancreatic Cancer UK (PCUK) |
| 23 | Dutch Pancreatic Cancer Group (DPCG) |
| 24 | Cancer Society of PALEMA (PALEMA) |
| 25 | Arbeitskreis der Pankreatekomierten (AdP) |
| 26 | Living with Hope (LWH) |
| 27 | Pankreaskreft Nettverk Norge (PKNN) |
| 28 | International Prehabiliation Society (iPOETTS) |
| 29 | United European Gastroenterology (UEG) |
| 30 | European Society for Medical Oncology (ESMO)** |

** Guideline observer members

**Table 2. Working group themes**

| WG | Topic |
| --- | --- |
| 1 | Early detection, imaging at diagnosis and staging |
| 2 | Biliary drainage/biopsy |
| 3 | Neoadjuvant and induction therapy |
| 4 | Preoperative counseling and prehabilitation |
| 5 | Surgery |
| 6 | Postoperative in-hospital management |
| 7 | External tumor directed percutaneous approaches |
| 8 | Adjuvant treatment |
| 9 | Palliative tumor-directed systemic treatment |
| 10 | Medical treatment |
| 11 | Palliative and supportive care |
| 12 | Pathology |
| 13 | Follow-up |

**Table 3.** GRADE levels of the certainty of evidence (43)

| Quality level | Definition |
| --- | --- |
| High | The true effect lies close to that of the estimated effect. |
| Moderate | The true effect is likely to be close to the estimated effect, but there is a possibility that it is substantially different |
| Low | The true effect might be substantially different from the estimated effect |
| Very low | The true effect is likely to be substantially different from the estimated effect |

**Figure 1**. Flow chart of guideline development stages

**Supplementary Table 1.** Participants in the development of the Guideline

|  | **Society** | **Member** | **Country** |
| --- | --- | --- | --- |
| 01 | E-AHPBA | 1.1 Marc Besselink | The Netherlands |
| 02 |  | 1.2 Thilo Hackert | Germany |
| 03 |  | 1.3 Bas Groot Koerkamp | The Netherlands |
| 04 |  | 1.4 Christoph Michalski | Germany |
| 05 |  | 1.6 Isabella Frigerio | Italy |
| 06 |  | 1.7 Giuseppe Malleo | Italy |
| 07 |  | 1.8 Giovanni Marchegiani | Italy |
| 08 |  | 1.9 Ugo Boggi | Italy |
| 09 | ESSO | 2.1 Elena Rangelova | Sweden |
| 10 |  | 2.2 Stefan Stättner | Austria |
| 11 |  | 2.3 Stefano Partelli | Italy |
| 12 |  | 2.4 Sven Mieog | The Netherlands |
| 13 |  | 2.5 Alain Sauvanet | France |
| 14 | ESGAR | 3.1 Marc Zins | France |
| 15 |  | 3.2 Nikolaos Kartalis | Sweden |
| 16 | EPC | 4.1 Hana Algül | Germany |
| 17 |  | 4.2 Livia Archibugi | Italy |
| 18 | ESDO | 5.1 Eric van Cutsem | Belgium |
| 19 |  | 5.2 Thomas Seufferlein | Germany |
| 20 |  | 5.3 Pieter-Jan Cuyle | Belgium |
| 21 | ISGPS | 6.1 Jens Werner | Germany |
| 22 |  | 6.2 Roberto Salvia | Italy |
| 23 | ESTRO | 7.1 Marta Scorsetti | Italy |
| 24 |  | 7.2 Somnath Mukherjee | United Kingdom |
| 25 |  | 7.3 Florence Huguet | France |
| 26 | ESP | 8.1 Caroline Verbeke | Norway |
| 27 |  | 8.2 Irene Esposito | Germany |
| 28 | ISGPP | 9.1 Arantza Farina Sarasqueta | The Netherlands |
| 29 |  | 9.2 Volkan Adsay | Turkey |
| 30 | ESGE | 10.1 Hannah van Malenstein | Belgium |
| 31 |  | 10.2 Geoffroy Vanbiervliet | France |
| 32 |  | 10.3 Simon Everett | United Kingdom |
| 33 | CIRSE | 11.1 Martijn Meijerink | The Netherlands |
| 34 |  | 11.2 Franco Orsi | Italy |
| 35 | E-CAPS | 12.1 Keith Roberts | United Kingdom |
| 36 |  | 12.2 Stefan Kobbelgaard Burgdorf | Denmark |
| 37 | ESRA | 13.1 Eleni Moka | Greece |
| 38 |  | 13.2 Thomas Volk | Germany |
| 39 | PCE | 14.1 Núria Malats | Spain |
| 40 |  | 14.2 Sorin Barbu | Romania |
| 41 | DiCE | 15.1 Zorana Maravic | Serbia |
| 42 |  | 15.2 Ana Martins | Belgium |
| 43 | EANM | 16.1 Cristina Nanni | Italy |
| 44 |  | 16.2 Valentina Ambrosini | Italy |
| 45 | SIOG | 17.1 Demetris Papamichael | Cyprus |
| 46 |  | 17.2 Gabor Liposits | Hungary |
| 47 | EAPC | 18.1 Anne Letsch | Germany |
| 48 |  | 18.2 Cathy Payne | Ireland |
| 49 | EONS | 19.1 Amanda Drury | Ireland |
| 50 |  | 19.2 Celia Diez de los Rios | Spain |
| 51 | EDS | 20.1 Dejan Radenkovic | Serbia |
| 52 |  | 20.2 Ilaria Pergolini | Germany |
| 53 | E-MIPS | 21.1 Mohammad Abu Hilal | Italy |
| 54 | PCUK | 22.1 Peter De Rosa | United Kingdom |
| 55 |  | 22.2 Alice Clarkson | United Kingdom |
| 56 | DPCG | 23.1 Hanneke Wilmink | The Netherlands |
| 57 |  | 23.2 Lois Daamen | The Netherlands |
| 58 | PALEMA | 24.1 Eva Backman | Sweden |
| 59 | AdP | 25.1 Matthias Seebo | Germany |
| 60 | LWH | 26.1 Pex Langenberg | The Netherlands |
| 61 | PKNN | 27.1 Charlotte Borge-Andersen | Norway |
| 62 | iPOETTS | 28.1 Marcel Den Dulk | The Netherlands |
| 63 |  | 28.2 Denny Levett | United Kingdom |
| 64 | UEG | 29.1 Giovanni Marchegiani | Italy |
| 65 |  | 29.2 Livia Archibugi | Italy |
| 66 |  | 29.3 Pieter-Jan Cuyle | Belgium |
| 67 | Researchers | 30.1 Laura Leeuwenburgh | The Netherlands |
| 68 |  | 30.2 Thomas Stoop | The Netherlands |
| 69 |  | 30.3 Ingmar Rompen | Germany |
| 70 |  | 30.4 Roberto Montorsi | Italy |
| 71 |  | 30.5 Luana Genova | Italy |
| 72 |  | 30.6 Benedict Kinny-Köster | Germany |
| 73 |  | 30.7 Riccardo Guastella | Italy |
| 74 |  | 30.8 Magdalena Holze | Germany |
| 75 |  | 30.9 Nuray Tezcan | Turkey |
| 76 |  | 30.10 Anne Gehrels | The Netherlands |
| 77 |  | 30.11 Charlotte Baggerman van Houweninge | The Netherlands |
| 78 |  | 30.12 Bas Uijterwijk | The Netherlands |
| 79 |  | 30.13 Gabriella Lionetto | Italy |
| 80 |  | 30.14 Fabio Casciani | Italy |
| 81 |  | 30.15 Ammar Javed | The Netherlands |
| 82 |  | 30.16 Manuela Mastronardi | Italy |
| 83 |  | 30.17 Amber Lamoré | The Netherlands |
| 84 |  | 30.18 Lis Hoeijmakers | The Netherlands |
| 85 |  | 30.19 Caro Bruna | The Netherlands |
| 86 |  | 30.20 Seng Liem | The Netherlands |
| 87 |  | 30.21 Alice Cattelani | Italy |
| 88 |  | 30.22 Carlo Maurer | Germany |
| 89 |  | 30.23 Ricardo Alvarez Jimenez | The Netherlands |
| 90 |  | 30.24 Lorenzo Lo Faro | Italy |
| 91 |  | 30.25 Jacobien Scheepens | The Netherlands |
| 92 |  | 30.26 Lisanne Lutter | The Netherlands |
| 93 |  | 30.27 Aniek van Diepen | The Netherlands |
| 94 |  | 30.28 Federica Vernuccio | Italy |
| 95 |  | 30.29 Dena Akhoundzadeh | The Netherlands |
| 96 |  | 30.30 Adnan Alsourani | Spain |
| 97 |  | 30.31 Jeska Fritzsche | The Netherlands |
| 98 |  | 30.32 Rogier Voermans | The Netherlands |
| 99 |  | 30.33 Heleen Driessens | The Netherlands |
| 100 |  | 30.34 Paul Andel | The Netherlands |
| 101 |  | 30.35 Roberta Vella | Italy |
| 102 |  | 30.36 Julian Musa | Germany |
| 103 |  | 30.37 Dana Sochorova | Czech Republic |
| 104 |  | 30.38 Berk Kaan Aktas | Turkey |
| 105 |  | 30.39 Alberto Balduzzi | Italy |
| 106 |  | 30.40 Mahsoem Ali | The Netherlands |
| 107 |  | 30.41 Eline van Gansewinkel | The Netherlands |
| 108 |  | 30.42 Gonzalo Gómez Dueñas | Spain |
| 109 |  | 30.43 Tijs Hoogteijling | The Netherlands |
| 110 | Methodologists | 31.1 Miranda Langendam | The Netherlands |
| 111 |  | 31.2 Yasaman Vali | The Netherlands |
| **Guideline observer members** | | | |
| 112 | ESMO | 32.1 Thierry Conroy | France |
| 113 |  | 32.2 Per Pfeiffer | Denmark |
| 114 |  | 32.3 Michel Ducreux | France |
